# Correction: Liquid biopsies for omics-based analysis in sentinel mussels

**DOI:** 10.1371/journal.pone.0225359

**Published:** 2019-11-12

**Authors:** France Caza, Philippine Granger Joly de Boissel, Richard Villemur, Stéphane Betoulle, Yves St-Pierre

The second author’s initials appear incorrectly in the citation. The correct citation is: Caza F, Granger Joly de Boissel P, Villemur R, Betoulle S, St-Pierre Y (2019) Liquid biopsies for omics-based analysis in sentinel mussels. PLoS ONE 14(10): e0223525. https://doi.org/10.1371/journal.pone.0223525
