# Supplementary figures and images for: Liquid biopsies for omics-based analysis in sentinel mussels
Source: PLoS One. 2019 Oct 3;14(10):e0223525. doi: 10.1371/journal.pone.0223525 (PMC6776352; doi:10.1371/journal.pone.0223525)

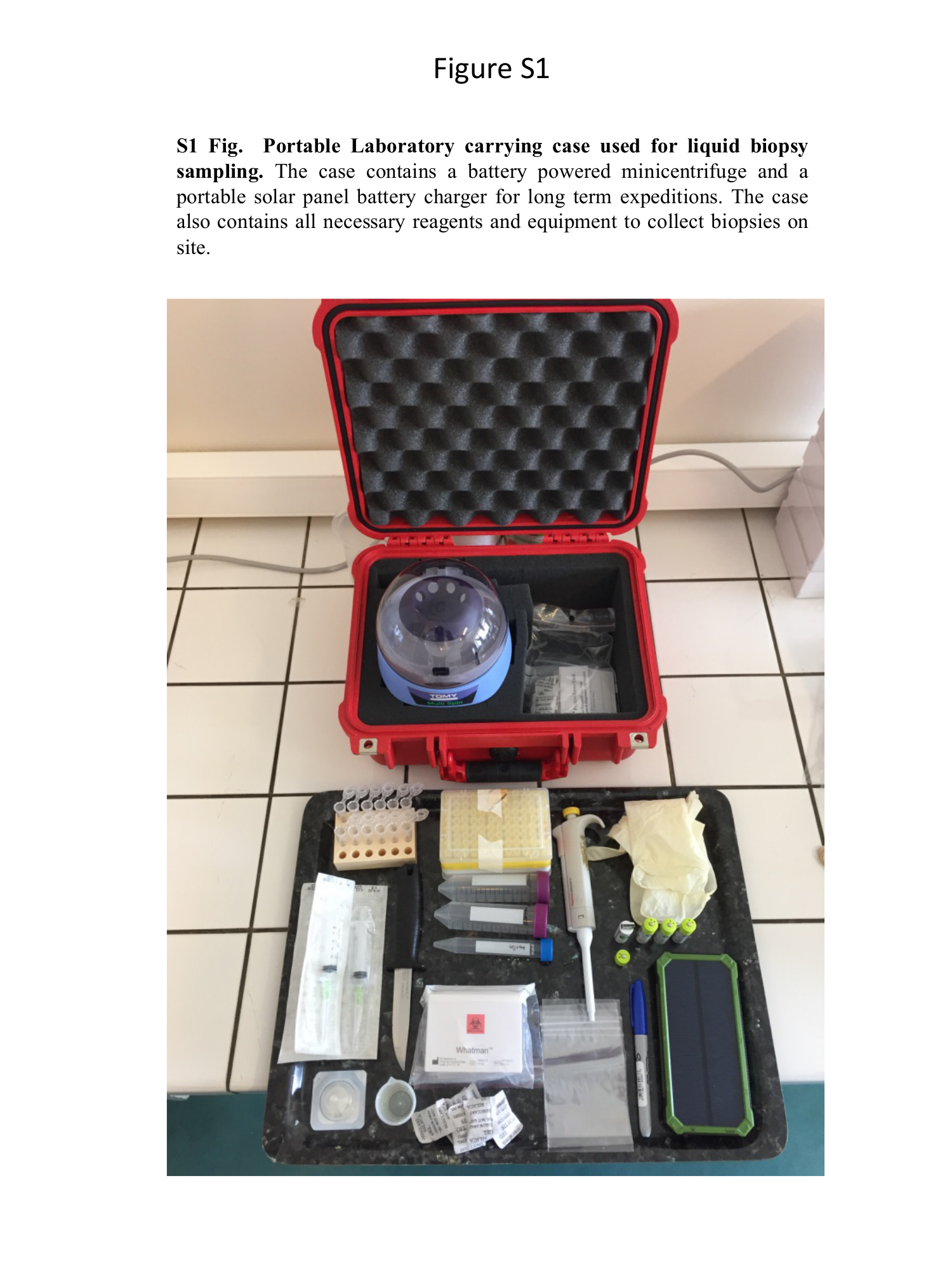

Supplement: S1 Fig — The case contains a battery powered minicentrifuge and a portable solar panel battery charger for long term expeditions. The case also contains all necessary reagents and equipment to collect biopsies on site. (TIFF) [file pone.0223525.s001.tiff]

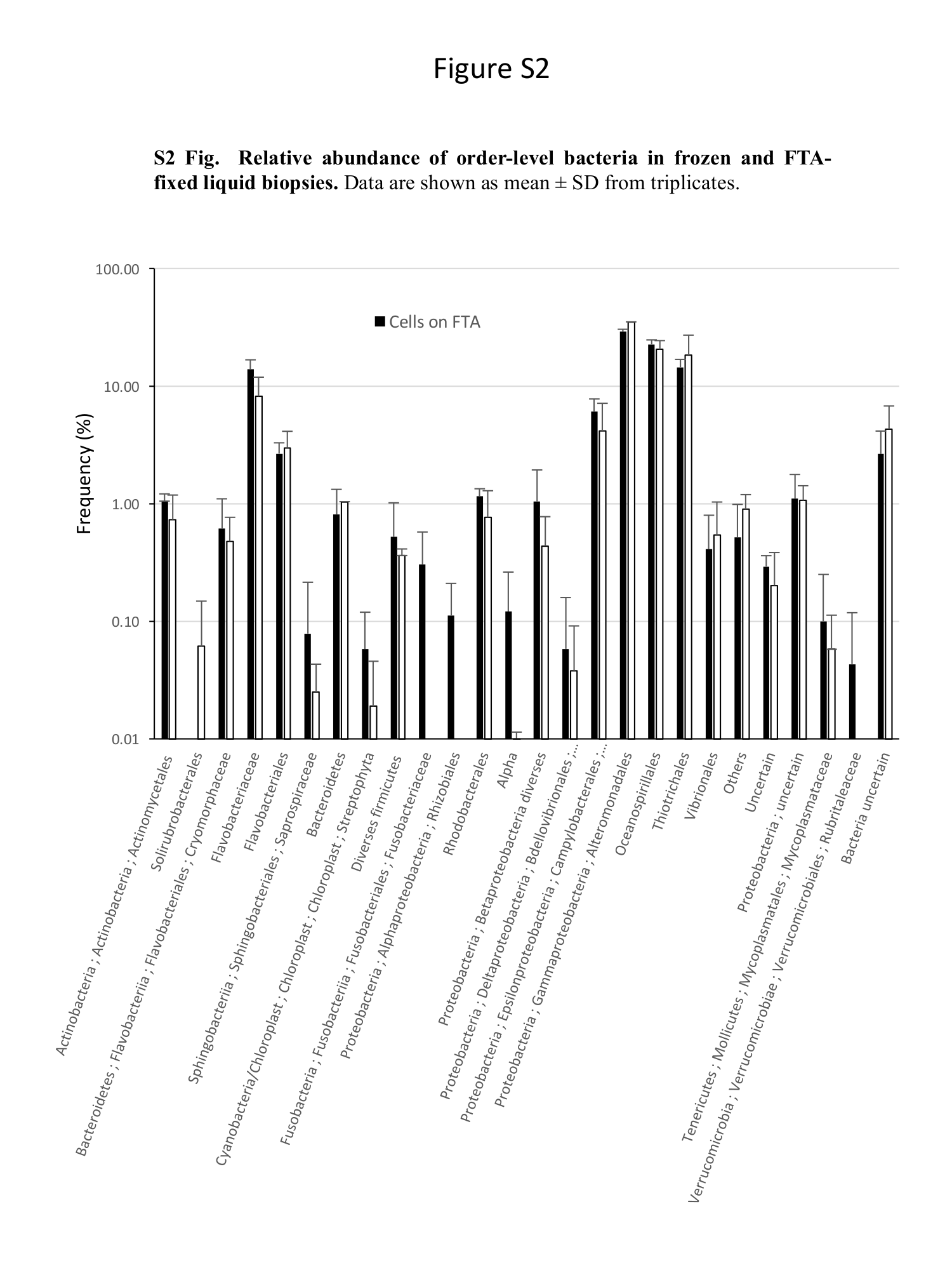

Supplement: S2 Fig — Data are shown as mean ± SD from duplicates. (TIFF) [file pone.0223525.s002.tiff]

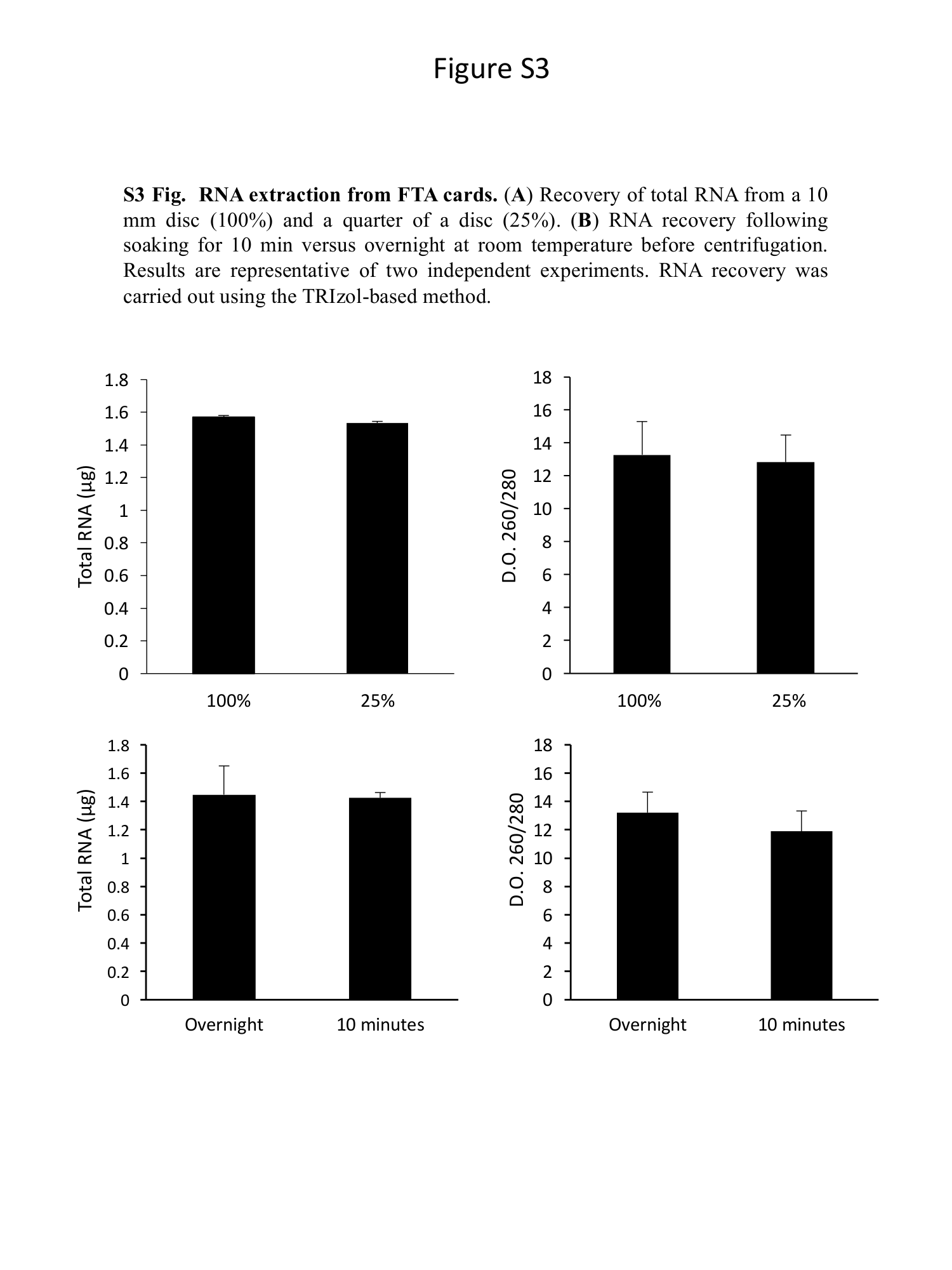

Supplement: S3 Fig — (A) Recovery of total RNA from a 10 mm disc (100%) and a quarter of a disc (25%). (B) RNA recovery following soaking for 10 min versus overnight at room temperature before centrifugation. Results are representative of two independent experiments. RNA recovery was carried out using the TRIzol-based method. (TIFF) [file pone.0223525.s003.tiff]

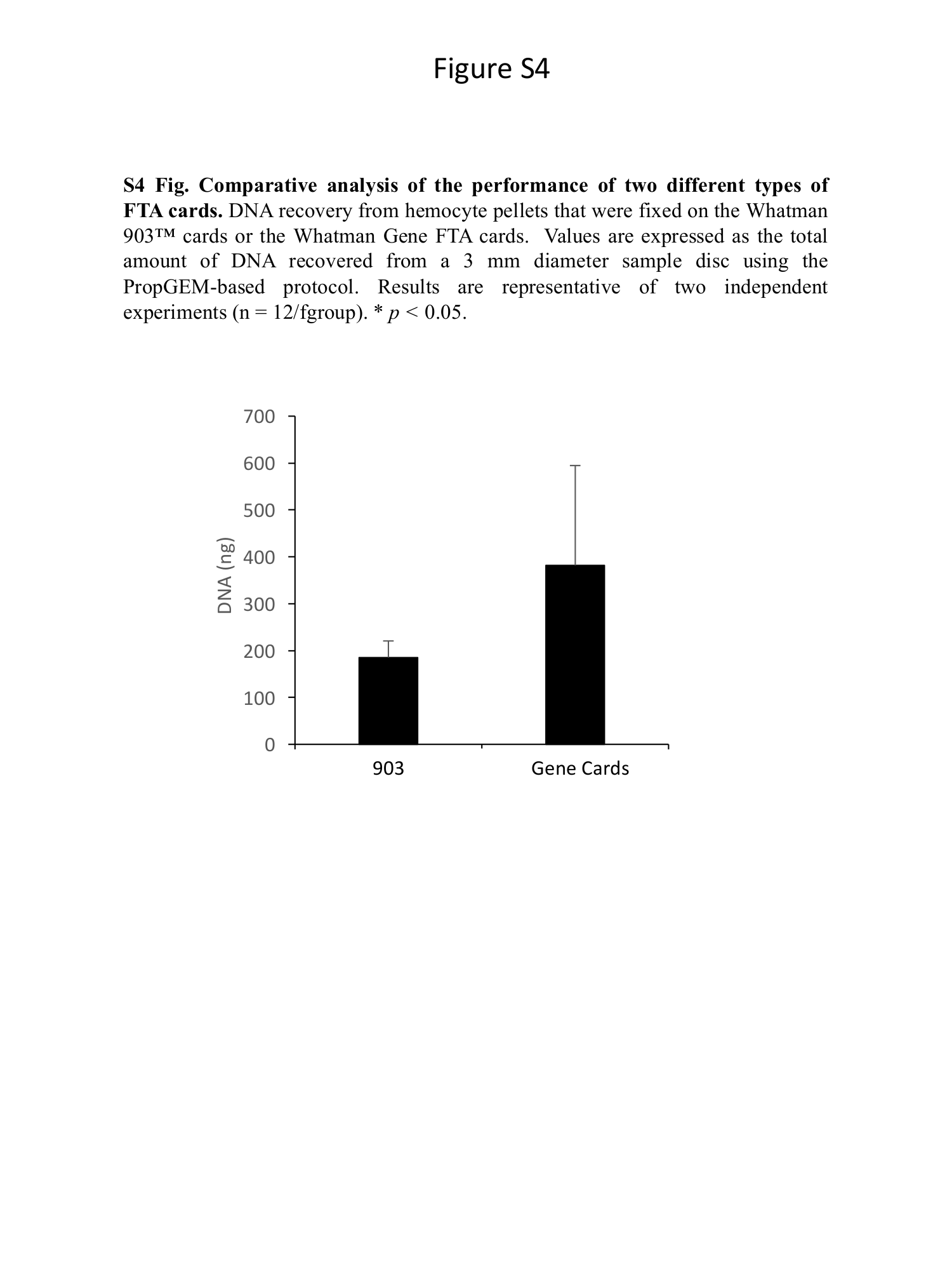

Supplement: S4 Fig — DNA recovery from hemocyte pellets that were fixed on the Whatman 903™ cards or the Whatman Gene FTA cards. Values are expressed as the total amount of DNA recovered from a 3 mm diameter sample disc using the PropGEM-based protocol. Results are representative of two independent experiments (n = 12/fgroup). * p < 0.05. (TIFF) [file pone.0223525.s004.tiff]
