# Supplementary material for: Liquid biopsies for omics-based analysis in sentinel mussels
Source: PLoS One. 2019 Oct 3;14(10):e0223525. doi: 10.1371/journal.pone.0223525 (PMC6776352; doi:10.1371/journal.pone.0223525)
Supplement: S1 Table — (DOCX) [file pone.0223525.s005.docx]

**Supplementary Table I:** RT-qPCR analysis of RNA extracted from frozen or FTA-fixed cell pellets

| Methodology | CT | MT |
| --- | --- | --- |
| RNAeasy mini kit | 13,46 ± 0,37 * | 83,38 ± 4,12 * |
| Trizol | 13,14 ± 0,39 * | 84,49 ± 0,05 * |
| No template control (NTC) | 22,89 ± 1,56 | 79,9 ± 0,14 |

Cycle threshold (Ct) and melting temperature (MT) obtained by RT-qPCR for *EF1 gamma* gene from FTA-fixed RNA extracted using the RNeasy Mini Kit from Qiagen and a standard organic extraction using Trizol extraction protocol. A non-template control (reaction mix with water) was used as control. (*p-value < 0.05; n= 5 with 3 replicates per sample; data shown as mean ± SD).
